# Mortality due to Sepsis and Its Associated Factors Among Patients Admitted to Intensive Care Units of Southern Amhara Public Hospitals, Ethiopia

**DOI:** 10.1155/2024/4378635

**Published:** 2024-10-28

**Authors:** Samuel Asmare Getu, Gebrehiwot Lema Legese, Kassahun Dessie Gashu, Desalew Getahun Ayalew, Tsegaw Amare Baykeda

**Affiliations:** ^1^Department of Nursing, Injibara Hospital, Injibara, Ethiopia; ^2^Department of Internal Medicine, School of Medicine, College of Medicine and Health Sciences, University of Gondar, Gondar, Ethiopia; ^3^Department of Health Informatics, Institute of Public Health, College of Medicine and Health Sciences, University of Gondar, Gondar, Ethiopia; ^4^Department of Health Systems and Policy, Institute of Public Health, College of Medicine and Health Sciences, University of Gondar, Gondar, Ethiopia; ^5^School of Public Health, The University of Queensland, Brisbane, Australia

**Keywords:** Ethiopia, intensive care units, public hospitals, sepsis, septic shock

## Abstract

**Introduction:** Although intensive care units (ICUs) are where severe and complicated cases are managed, there is limited evidence on treatment outcomes in Ethiopia. Therefore, this study is aimed at assessing the magnitude and associated factors of mortality among patients with sepsis admitted to ICUs at southern Amhara public hospitals, Ethiopia.

**Methods:** A total of 547 medical records of patients with sepsis admitted to the ICUs at Injibara, Debre Markos, and Debre Tabor hospitals in the past 3 years were retrieved from August 10–31, 2022. Multivariable logistic regression analyses were conducted and adjusted odds ratios (AOR) with a 95% confidence interval (CI) were reported, and a *p* value < 0.05 was set to declare the significance of the association.

**Results:** In this study, 46.2% (95% CI: 41.7%–50.3%) of patients with sepsis died during their ICU stay. Respiratory, gastrointestinal, and urinary tract infections contributed to 32.3%, 25.8%, and 9.6% of mortality, respectively. Individuals aged 60 and above (AOR: 4.07; 95% CI: 2.23–7.44), those with a Glasgow Coma Scale ≤ 10 at admission (AOR: 11.27; 95% CI: 4.64–27.37), respiratory site of infection (AOR: 5.38; 95% CI: 2.94–9.86), creatinine level > 1.1 mg/dL (AOR: 4.20; 95% CI: 2.33–7.60), vasopressor use (AOR: 3.13; 95% CI:1.66–5.95), initiation of antibiotics 1–3 h after admission (AOR: 2.80; 95% CI: 1.64–4.76), and a hospital stay of more than 20 days (AOR: 3.44; 95% CI: 1.40–8.46) were significantly associated with mortality.

**Conclusion:** Overall, the mortality rate among patients with sepsis admitted to ICUs in southern Amhara public hospitals was high. Mainly, death was attributed to respiratory infections. Elderly patients and those with deteriorated clinical conditions at admission were at higher risk. Therefore, special attention is needed for elderly patients admitted with respiratory infections, antibiotics should be initiated as early as possible, and interventions must be designed to shorten the length of stay in ICUs.

## 1. Background

Sepsis is a life-threatening systemic inflammatory condition caused by a dysregulated host response to infection, leading to severe organ dysfunction [1]. Globally, 50 million people develop sepsis worldwide, and 11 million patients die due to sepsis annually [2]. Low and middle-income countries account for nearly 85% of cases of sepsis and 3.5 million mortality reported in sub-Saharan Africa in 2017 [3].

An intensive care unit (ICU) is a specialized unit in a hospital where patients requiring prolonged stays and complex therapies are admitted and treated. Despite advances in medical care, sepsis remains a significant cause of ICU admissions and mortality [3–5]. For example, studies conducted in high-income countries [6–10] have shown that mortality rates for sepsis patients admitted to ICUs range from 18.4% to 34.9%. Additionally, 17.5%–40.4% of patients with sepsis die in the ICU [11, 12]. Moreover, in Ethiopia, the mortality rate of patients with sepsis admitted to ICUs ranges from 41.8% to 44% [13, 14].

Different factors contribute to the mortality rate of sepsis patients in the ICUs. For instance, sociodemographic factors such as sex [15] and age [3] are associated with the mortality of sepsis patients in the ICU. Additionally, clinical-related factors such as infection sites [16–18], underlying comorbidity [2, 6, 13, 14, 19–21], time of initiation of antimicrobial therapy [14, 22], and time to use renal replacement therapy [21, 23] are associated with mortality among patients with sepsis in the ICU.

In the evolving clinical environment, various factors are associated with the mortality rate of sepsis patients in the ICU. However, limited studies have identified factors contributing to the mortality of sepsis patients in ICUs in Ethiopia. Although two studies [13, 24] have been conducted in this area, none have specifically assessed the magnitude of mortality among patients with sepsis in our study settings. Evidence in this group of patients will assist in decision-making for critically ill patients in the ICU including sepsis patients. Additionally, including multiple sites will enhance the representativeness of our findings. Furthermore, informs policymakers about preventable factors in the ICU and might be used as a baseline finding to conduct further studies. Therefore, this study is aimed at assessing the magnitude and associated factors of mortality among patients with sepsis admitted to the ICUs of public hospitals in southern Amhara, Ethiopia.

## 2. Methods

### 2.1. Study Design and Settings

A hospital–based retrospective follow-up study was conducted among patients diagnosed with sepsis and admitted to ICUs from 2020 to 2022, at public hospitals located in the southern part of the Amhara region. The Amhara region is one of Ethiopia's federal regional states, with its capital in Bahirdar. As of 2022, the region is estimated to have a population of 30,848,988, with 59% comprising men and 88% residing in rural areas [25]. The region has 83 hospitals, with nine public hospitals equipped with ICUs. For this study, we specifically included three public hospitals with ICUs in the southern part of the region: Injibara Hospital, Debre Markos Hospital, and Debre Tabor Hospital. The Ethiopian healthcare system operates under a three-tier healthcare delivery, with referral hospitals expected to provide comprehensive healthcare services to critically ill patients. According to Ethiopian standards for referral hospitals, ICUs must be well-equipped to offer optimal life support and ensure adequate monitoring of vital functions for seriously ill patients [26].

### 2.2. Source and Study Population

The source population for this study comprised all adult patients (≥ 18 years of age) diagnosed with sepsis and admitted to ICUs at the three public hospitals. The study population included all selected adult patients diagnosed with sepsis and admitted to ICUs at these hospitals. However, patients who were referred to other hospitals were excluded from the study.

### 2.3. Sample Size Determination and Sampling Technique

The sample size for this study was determined using the single population proportion formula, considering a 95% confidence level, a 5% margin of error, and a proportion of 50.9% from a previous study conducted in Addis Ababa, Ethiopia [13]. With a design effect of 1.5 and a 10% nonresponse rate taken into account, the initial total sample size was calculated to be 634. However, since the total population size is less than 10,000, an adjustment formula was applied, resulting in a final sample size of 505.

Since the total number of patients with sepsis admitted to ICUs of the three hospitals in the past 3 years was 547, which is close to the sample size calculated for this study, we decided to employ a census of the study population at the three public hospitals. Therefore, all eligible 547 cases (194 samples from Injibara, 249 samples from Debre Markos, and 104 samples from Debre Tabor referral hospitals) of patients with sepsis were included.

### 2.4. Study Variables

The outcome variable of this study was death among sepsis-diagnosed patients admitted to ICUs, as recorded on patients' charts at the time of discharge, categorized as either “improved” or “died.” In this study, a patient admitted to the ICU with sepsis was considered “died” only if the attending physician of the ICU declared “expired” on the patient's chart.

The independent variables included sociodemographic factors such as age, sex, residence, and referral facility of the patient, as well as clinical characteristics such as vital signs, underlying comorbidities, site of infection, Glasgow Coma Scale (GCS), creatinine level, and length of hospitalization.

### 2.5. Data Collection Tools and Data Quality Control

We conducted a pretest of the tool on 27 (5%) charts of patients with sepsis at the University of Gondar Comprehensive Specialized Hospital ICU, and necessary modifications were made based on the results. The pretested and structured questionnaire was then used for data extraction by two BSc nurse professionals, supervised by one MSc holder in each hospital. The cases spanning 3 years were retrieved from the hospital archives using the department's logbook and medical records. To maintain data quality, the instrument was prepared in English. A day of training was provided for data collectors and a supervisor on the objectives of the study, the confidentiality of information, the relevance of the study, and technical aspects. Close supervision was maintained throughout the data collection period.

### 2.6. Data Management and Analysis

The completed data were cleaned and coded in EpiData Version 4.6 and exported to Stata Version 14 [27] for further analysis. We computed descriptive statistics, including frequencies, means with standard deviations (SDs), and percentages, which were presented through text, tables, and graphs. We checked the assumptions for the chi-square test, ensured multicollinearity with a variance inflation factor (VIF) of 1.25, and assessed the model's fitness using the Hosmer–Lemeshow goodness-of-fit test.

Both bivariable and multivariable logistic regression analyses were employed to identify associated factors. Variables significant at a *p* value < 0.25 in the bivariable logistic regression analysis were entered into the multivariable logistic regression model to control for confounding effects. Adjusted odds ratios (AORs) with 95% confidence intervals (CI) were computed to evaluate the strength of association and variables with a *p* value less than 0.05 were declared statistically significant factors associated with the outcome variable.

Furthermore, for logical and scientific representation of the study findings, we adhered to the guidelines outlined in the “Strengthening the Reporting of Observational Studies in Epidemiology (STROBE).” This ensured clarity and transparency in reporting our observational study [28].

### 2.7. Ethical Considerations

To access the data from medical records, ethical clearance was obtained, and the requirement for informed consent was waived by the Ethical Review Board of the School of Medicine, College of Medicine and Health Sciences, University of Gondar, with Reference No.: SOM/1801/22, granted on August 1, 2022. Additionally, strict confidentiality measures were adhered to, ensuring that medical records were not exposed to any third parties without authorization. Permission to access the medical records was also obtained from the head of the hospital's ICU. Furthermore, a supporting letter was obtained from the Amhara Public Health Institute, Reference No.: APHI 03/1542/14, on August 10, 2022. All methods were conducted following relevant guidelines and regulations to ensure ethical standards were maintained throughout the study.

## 3. Results

### 3.1. Sociodemographic Characteristics of Study Participants

Among the 547 patients with sepsis whose medical registration cards were reviewed, 511 (93.4%) were included in the study after meeting the eligibility criteria. Twenty charts did not report the treatment outcome, and 16 charts indicated that patients were transferred to another facility. The median age of the patients was 44 years, with an interquartile range of 25–75. Notably, 43.4% of the patients were aged between 18 and 39 years. In terms of gender distribution, more than half (55.8%) of the patients admitted to ICUs with sepsis were females. Additionally, higher proportions (80%) of the patients were rural residents (Table 1).

### 3.2. Clinical Characteristics of the Study Participants

**Figure 1:**
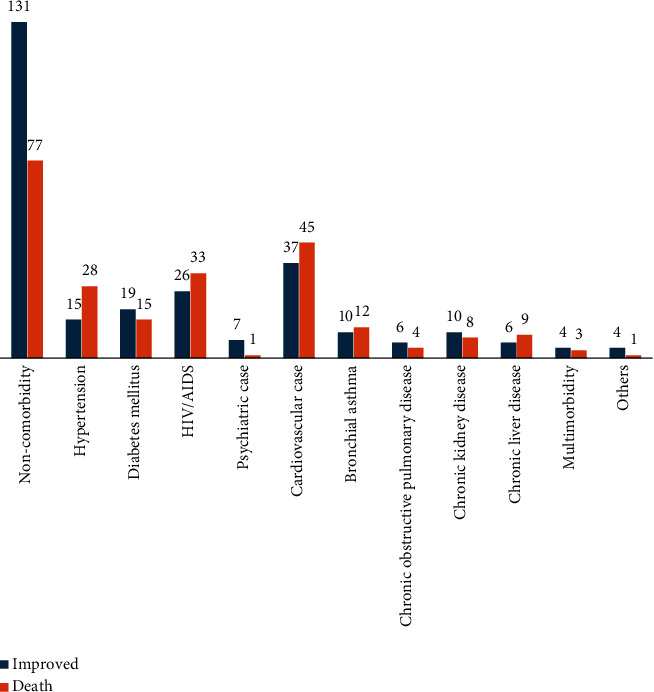
Comorbidity among patients with sepsis admitted to ICUs of southern Amhara public hospitals, Ethiopia.

**Figure 2:**
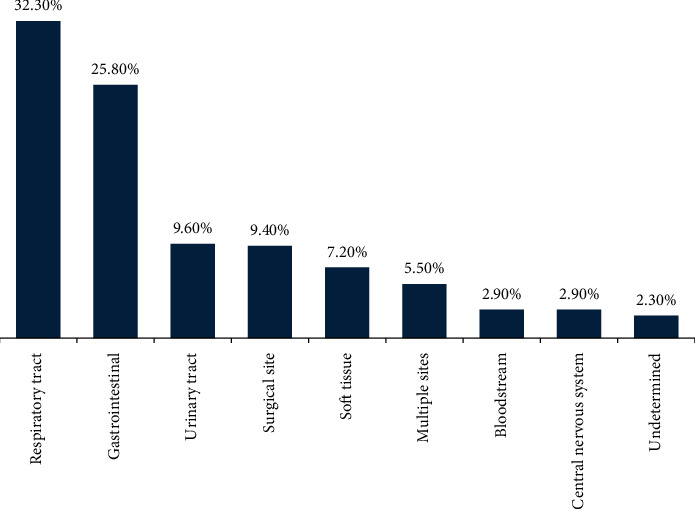
Primary focus of infection among patients with sepsis admitted to ICUs of southern Amhara public hospitals, Ethiopia.

In this study, 319 (62.4%) of the patients were febrile (> 37.5°C) while admitted to ICUs and more than half 275 (53.8%) of them had a GCS score between 10 and 13 scores (Table 2). More than half 303 (59.3%) of the patients had one or more comorbidity identified during admission (Figure 1). Besides, the primary focus of patients with sepsis was respiratory 165 (32.30%), followed by gastrointestinal 132 (25.80%), and 28 (5.50%) patients had more than one focus of infection (Figure 2).

In this study, the magnitude of mortality among patients with sepsis admitted to ICUs of southern Amhara public hospitals was 236 (46.2%; 95% CI: 41.7%–50.3%).

### 3.3. Factors Associated With the Magnitude of Mortality Among Patients With Sepsis Admitted to ICUs of Southern Amhara Public Hospitals, Ethiopia

In the bivariable logistic regression analysis, several factors including age, residence, referral status, body temperature during admission, GCS level during admission, creatinine level during admission, primary focus of infection, blood transfusion, vasopressor use, antibiotic starting time, comorbidity, and length of hospitalization had a *p* value < 0.25 and were considered candidates for multivariable logistic regression analysis.

In the multivariable logistic regression analysis, age, GCS level during admission, creatinine level during admission, primary focus of infection, blood transfusion, vasopressor use, antibiotic starting time, and length of hospitalization were found to be significantly associated with mortality, with a *p* value < 0.05.

Specifically, the odds of mortality among patients aged 60 and above were 4.07 (95% CI: 2.23–7.44) times compared to patients aged 18–39 years. Patients with a GCS score of ≤ 10 at admission had a probability of mortality of 11.27 (95% CI: 4.64–27.37) times than those with a GCS score ≥ 14 at admission. Furthermore, patients with a creatinine level ≥ 1.1 mg/dL at admission had 4.20 (95% CI: 2.33–7.60) times the odds of mortality compared to those with a creatinine level < 1.1 mg/dL.

Patients with a respiratory focus of infection were 5.38 (5.38; 95% CI: 2.94–9.86) times the odds of mortality compared to those with other sites of infection. Additionally, the likelihood of mortality among patients who received vasopressors was 3.13 (95% CI: 1.66–5.95) times compared to those who did not. Patients who initiated antibiotic treatment within 1–3 h had 2.80 (95% CI: 1.64–4.76) times the odds of mortality compared to those who started antibiotics within less than 1 h. Moreover, patients with a hospital stay of more than 20 days had 3.44 (95% CI: 1.40–8.46) times the odds of mortality compared to those with a stay of less than 10 days (Table 3).

## 4. Discussion

This study revealed that nearly half of the patients with sepsis admitted to ICUs in a public hospital in the southern part of the Amhara region died between 2020 and 2022. Factors such as the age of the patient, the clinical condition at admission, the site of infection, vasopressor use, the timing of antibiotic initiation, and the length of hospital stay were identified as significant factors associated with death due to sepsis in ICUs.

In this study, nearly half (46.2%) of the patients with sepsis admitted to ICUs of Injibara, Debre Markos, and Debre Tabor hospitals lost their lives. This is consistent with the finding of the study conducted at the University of Gondar Hospital, where the death rate of patients in the ICU was 42% [14]. However, lower numbers (26.5%) of patients with sepsis admitted to ICUs died in Addis Ababa [13]. This might be due to the difference in the quality of ICU service, availability of medications such as antibiotics, and access to infrastructures such as mechanical ventilation [29]. The finding is higher than the 39.70% pooled magnitude of ICU death in Ethiopia [30]. This might be because of the devastating and acute complications of sepsis compared to other cases [31] and might imply the worst-case survival rate in the study settings urging attention from the officials. Our finding is also higher than the studies conducted in Zambia [11] and China [7], where 40% and 13.1% of the patients admitted to ICUs have died. In addition, this finding is higher than the study conducted in European ICUs, where only 10%–38% of the patients with sepsis admitted at ICUs lost their [32]. This discrepancy might be due to the difference in the study settings, lifestyle of the patient, patient care, and treatment setup.

This study also revealed that more than half of patients who died due to sepsis had a respiratory primary focus of infection followed by a gastrointestinal focus of infection. This is also illustrated in our regression analysis that patients who had the respiratory and gastrointestinal focus of infection were more likely to die due to sepsis compared to other sites of infection, respectively. This is in line with the study conducted in Addis Ababa, where 53.1% of deaths due to sepsis originated from respiratory infection [13], but contrary to the study conducted at the University of Gondar Hospital, where the leading cause of sepsis death in ICUs was gastrointestinal (61.2%) followed by respiratory (30.6%) infections [14]. The finding also exhibits similarity with the study conducted in China, where 34.7% of mortality due to sepsis in ICUs was among patients with a pulmonary focus on infection [7]. The studies conducted elsewhere [5, 7, 8, 13, 14, 16, 17] support the findings of this study.

Moreover, this study illustrated that the odds of death among elderly patients were higher compared to young patients. It parallels the findings of the studies conducted in the rest of the world [2, 3, 10, 15, 18, 33, 34]. This is consistent with the scientifically proven risk of higher age for immune compromise [35, 36]. However, the study at the University of Gondar Hospital indicated mortality due to sepsis had no significant association with the age of the patient [14]. The reason might be due to the small sample size and potential selection bias of the study conducted at the University of Gondar Hospital as the incomplete medical record was recorded as the limitation of the study [14]. In addition, contrary to the study at the University of Gondar Hospital [14], the odds of death among patients with sepsis who had used vasopressor were tripled compared to their counterparts. But it is in line with the studies conducted elsewhere [7, 37, 38]. The possible reason might be that most of the patients start management for vasopressor whenever they are at the end stage of their life. At that time, the outcome might be poor because the patient might have organ failure and other underlying conditions against the treatment.

This study revealed that patients presenting to the ICUs with debilitating clinical conditions will probably lose their lives due to sepsis. This is supported by the study conducted in Zambia [11]. The possible explanation might be due to most patients with sepsis are admitted to ICUs when getting critically ill so the outcome will be poor because the patient might have organ failure, cardiogenic shock and unknown other underlying health problems. Besides, the likelihood of death among patients who had higher creatinine levels at admission was higher compared to patients who had lower creatinine levels. This might be due to the reduced energy production and metabolic rate because of hormonal and inflammatory mediators leading to acute kidney injury or renal failure [39].

Furthermore, this study exhibited that late initiation of antibiotics and a long stay at ICUs were associated with a higher probability of death due to sepsis. It is consistent with the study conducted in China [7]. This underlines that early diagnosis and treatment with antibiotics might improve the patient's clinical conditions. Besides, studies conducted elsewhere [2, 14, 21, 22] supported the findings that a long stay in the ICU increases the probability of death from the disease. This might be attributable to the development of a complication, hospitalization-induced stress, and additional hospital-acquired infection contributed to mortality.

This study included patients from three different hospitals to increase their representativeness in similar settings. However, several limitations should be taken into consideration. Firstly, this study could not determine the exact cause of death of the patients, as several factors, such as delays in presentation to the ICU, which we might not have included, could contribute to it. Secondly, reliance on medical records as the primary data source may result in the omission of crucial variables, such as facility-related and behavioural factors, potentially weakening the model's predictive power. Incorporating additional data sources or employing alternative methodologies could address this limitation. Finally, the institution-based nature of the study may limit the generalizability of our findings to the broader community. Future research could benefit from including a more diverse range of healthcare facilities and patient populations to enhance the external validity of the study findings.

## 5. Conclusion

In conclusion, the study highlights a high mortality rate among patients with sepsis admitted to ICUs, higher than the pooled prevalence of ICU death in Ethiopia. Respiratory and gastrointestinal infections were identified as leading causes of death, emphasizing the need for targeted interventions. Elderly patients, those with deteriorated clinical conditions at admission, respiratory site of infection, vasopressor use, delayed initiation of antibiotics, and prolonged hospital stays were identified to be associated with the death of patients diagnosed with sepsis in the ICU.

Hence, early initiation of antibiotics and shortening the stay of patients in ICU might save the patients' lives. Additionally, specialized care and critical follow-up for elderly patients admitted with respiratory infections are essential. Given the critical nature of the issue, further studies, such as implementation research, should be conducted to uncover the practical challenges of sepsis patients admitted to ICUs.

## Figures and Tables

**Table 1:** Sociodemographic characteristics of patients with sepsis admitted to intensive care units of public hospitals in Amhara region, Ethiopia, 2022 (*N* = 511).

**Variables**	**Category**	**Frequency**	**Percent**
Age at diagnosis (years)	18–39	222	43.5
	40–59	157	30.7
	≥ 60	132	25.8

Sex of the patient	Male	226	44.2
	Female	285	55.8

Residency	Rural	409	80.0
	Urban	102	20.0

Referred from	Health center	230	45.0
	Clinics and hospitals	209	40.9
	Self	72	14.1

**Table 2:** Clinical characteristics of patients with sepsis admitted to intensive care units of public hospitals in Amhara region, Ethiopia, 2022 (*N* = 511).

**Variables**	**Category**	**Frequency**	**Percent**
Body temperature at admission (°C)	< 35.5	74	14.5
	35.5–37.5	118	23.1
	> 37.5	319	62.4

White Blood Cell count at admission (per litre)	< 4.5⁣^∗^10^9^	57	11.2
	4.5-11.0⁣^∗^10^9^	105	20.5
	> 11.0⁣^∗^10^9^	342	66.9
	Unknown	7	1.4

Creatinine level at admission (mg/dl)	< 1.1	293	57.3
	≥ 1.1	128	25.0
	Unknown	90	17.6

Glasgow Coma Scale at admission	≥ 14	100	19.6
	11-13	275	53.8
	≤10	136	26.6

Vasopressor used	Yes	401	78.5
	No	110	21.5

Number of vasopressors used	Single	338	66.1
	≥ 2	63	12.3

Length of vasopressor used (hours)	< 6	144	28.2
	6–11	208	40.7
	≥ 12	49	9.6

Blood transfused	Yes	109	21.3
	No	402	78.7

Antibiotic initiation time (hours)	< 1	150	29.4
	1–3	328	64.2
	> 3	33	6.5

Steroids used	Yes	393	76.9
	No	118	23.1

Presence of comorbidity	Yes	303	59.3
	No	208	40.7

Intubated	Yes	334	65.4
	No	177	34.6

Length of intubation (days)	1–3	275	53.8
	4–8	46	9.0
	> 8	13	2.5

Cardioversion used	Yes	31	6.1
	No	480	93.9

Length of hospitalization (days)	1–10	325	63.6
	11–20	146	28.6
	> 20	40	7.8

**Table 3:** Bivariable and multivariable logistic regression analysis of factors associated with mortality among patients with sepsis admitted to intensive care units of public hospitals in Amhara region, Ethiopia, 2022.

**Variables**	**Outcome**		**COR (95% CI)**	**AOR (95% CI)**
	**Death**	**Improve**		
Age at diagnosis (years)				
18–39	72	150	1	1
40–59	71	86	1.72 (1.13, 2.62)	1.44 (0.83, 2.48)
≥ 60	93	39	4.97 (3.11, 7.93)	**4.07 (2.23, 7.44)**⁣^∗∗^
Residency of the patient				
Rural	180	229	0.65 (0.42, 1.0)	0.69 (0.36, 1.34)
Urban	56	46	1	1
Referred from				
Health center	98	132	0.56 (0.33, 0.96)	1.25 (0.54, 2.90)
Clinics and hospitals	97	112	0.66 (0.38, 1.12)	1.35 (0.59, 3.08)
Self	41	31	1	1
GCS at admission				
≥ 14	15	85	1	1
10–13	132	143	5.23 (2.88, 9.51)	**9.87 (4.32, 22.57)**⁣^∗∗^
< 10	89	47	10.73 (5.59, 20.61)	**11.27 (4.64, 27.37)**⁣^∗∗^
Body temperature at admission (^0^c)				
<35.5	53	21	4.92 (2.61, 9.27)	1.06 (0.58, 1.95)
35.5–37.5	40	78	1	1
>37.5	143	176	1.58 (1.02, 2.46)	2.28 (0.97, 5.33)
Creatinine level at admission (mg/dl)				
<1.1	106	187	1	1
>1.1	87	41	3.68 (2.35, 5.75)	**4.21 (2.33, 7.59)**⁣^∗∗^
Unknown	43	47	1.59 (0.98, 2.57)	1.47 (0.78, 2.74)
Primary focus of infection				
Respiratory	95	70	3.54 (2.23, 5.60)	**5.38 (2.94, 9.86)**⁣^∗∗^
Gastrointestinal	68	64	2.77 (1.71, 4.48)	**5.18 (2.68, 9.99)**⁣^∗∗^
Surgical	27	21	3.35 (1.72, 6.51)	**4.55 (1.82, 11.34)**⁣^∗^
Others⁣^∗^	46	120	1	
Vasopressor used				
Yes	207	194	2.98 (1.87, 4.76)	**3.13 (1.66, 5.95)**⁣^∗∗^
No	29	81	1	1
Blood transfused				
Yes	60	49	1.57 (1.03, 2.41)	1.77 (0.98, 3.19)
No	176	226	1	1
Antibiotic starting time after admission (hours)				
< 1	48	102	1	1
1–3	169	159	2.26 (1.51, 3.39)	**2.80 (1.64, 4.76)**⁣^∗∗^
> 3	19	14	2.88 (1.33, 6.23)	1.39 (0.49, 3.64)
Comorbidity				
Yes	159	144	1.88 (1.31, 2.69)	1.41 (0.88, 2.28)
No	77	131	1	1
Stay of hospitalization (days)				
1–10	114	211	1	1
11–20	96	50	3.55 (2.36, 5.36)	**3.87 (2.26, 6.65)**⁣^∗∗^
> 20	26	14	3.44 (1.72, 6.84)	**3.44 (1.40, 8.46)**⁣^∗^

*Note:* All boldfaced results are significant values.

⁣^∗∗^Significant at *p* value < 0.01.

⁣^∗^Significant at *p* value < 0.05.

## Data Availability

The datasets used and/or analyzed during the current study are available from the corresponding author upon reasonable request.
